# Schulprogramme zur Förderung der psychischen Gesundheit. Die psychische Gesundheitskompetenz von Lehrpersonen als wichtiger Umsetzungsfaktor

**DOI:** 10.1007/s11553-022-01008-1

**Published:** 2023-01-16

**Authors:** Dominik Robin, Kurt Albermann, Julia Dratva

**Affiliations:** 1 Institut für Public Health, Department Gesundheit ZHAW, Winterthur, Schweiz; 2 Sozialpädiatrisches Zentrum SPZ, Kantonsspital Winterthur, Winterthur, Schweiz

**Keywords:** Gesundheitskompetenz, Pädagogische Fachpersonen, Schule, Psychische Belastung, Prävention, Kinder, Health literacy, Pedagogical professionals, School, Mental health disorders, Prevention, Children

## Abstract

**Hintergrund:**

Weltweit sind bis zu einem Fünftel der Kinder und Jugendlichen von psychischen Störungen betroffen. Auffälligkeiten, die bereits im Schulalter auftreten, sind insbesondere für die Betroffenen, aber auch die Lehrpersonen belastend. Schulische Präventionsprogramme zur psychischen Gesundheit haben das Potenzial, die Belastungen beidseitig zu reduzieren. Die psychische Gesundheitskompetenz von Lehrpersonen, die nicht nur das Wissen und das Verständnis, sondern auch die Handlungen untersucht, gilt dabei als wichtiger Umsetzungsfaktor. Die Studienlage ist mager.

**Ziele:**

Die Studie untersucht die psychische Gesundheitskompetenz von Lehrpersonen im Kontext von Belastungssituation der Schülerinnen und Schüler sowie das Vorhandensein entsprechender Schulprogramme.

**Methoden:**

Im Schuljahr 2019/2020 wurden alle Lehrpersonen in einer Deutschschweizer Stadt zu einem Online-Survey eingeladen (*n* *=* *1514*; Rücklauf: 38 %). Die Daten wurden quantitativ mittels bivariater und multivariater Methoden ausgewertet. Eine offene Frage zum Verständnis psychischer Störungen wurde qualitativ mittels einer Inhaltsanalyse ausgewertet.

**Resultate und Diskussion:**

Die Befragten betreuten oder unterrichteten im letzten Jahr durchschnittlich 4,7 psychisch belastete Schülerinnen und Schülern. Die Anzahl Betroffener unterschied sich nach Schulstufe und Schulfunktion. Die Lehrpersonen schätzten ihre Fähigkeit, Informationen zum Thema zu finden und zu verstehen als (sehr) gut ein, es fiel ihnen jedoch schwerer, zu beurteilen, ob die gefundenen Informationen korrekt sind. Die Handlungskompetenz der Lehrkräfte war partiell gering, beispielsweise beim Wissen über Störungsbilder und stellt ein Hindernis in der Umsetzung von Schulprogrammen dar. Lehrkräfte relativierten auffälliges Verhalten, was die Triage für weitere Abklärungen erschwert. Den Schulen wird zum einen empfohlen in die Eigeninitiative der Lehrpersonen zu investieren, zum anderen sollte die Steigerung der psychischen Gesundheitskompetenz nicht nur Aufgabe der Lehrpersonen sein, sondern durch eine entsprechende Schulkultur und gesellschaftlich gefördert werden.

## Abstract (en)

**Background:**

Worldwide, up to one fifth of children and adolescents are affected by mental disorders. Disorders that occur as early as school age are particularly stressful for those affected, but also for teachers. School-based mental health prevention programs have the potential to reduce the burden on both sides. Mental health literacy of teachers, which not only investigates the knowledge and the understanding, but also action competencies, is considered to be an important factor of implementation. Studies in this area are scarce.

**Objectives:**

This study investigates mental health literacy of teachers in the context of school children’s stress levels and the existence of corresponding school programs.

**Methods:**

In the school year 2019/2020, all teachers of a German-speaking Swiss city were invited to an online survey (*N* = 1514*;* response rate: 38%). Data were evaluated quantitatively using bivariate and multivariate methods. An open question on the understanding of mental disorders was analyzed qualitatively with a content analysis.

**Results and conclusion:**

Participants supervised or taught an average of 4.7 mentally distressed school children in the past year. The number varied by school level and school function. Participants rated their ability to find and understand information on the topic as (very) good, but they found it more difficult to judge whether the information they found was correct. The teachers’ action competence was partially low, for example in terms of knowledge of mental disorders, and it represents an obstacle in the implementation of school programs. Teachers relativized conspicuous behavior, which makes triage for further medical clarification more difficult. On the one hand, it is recommended that schools invest in teachers’ own initiative, and on the other hand, increasing mental health literacy should not only be the task of teachers, but also be encouraged by an appropriate school culture and by society.

## Hintergrund und Fragestellung

Internationalen Studien zufolge sind weltweit etwa 10–20 % der Kinder und Jugendlichen von psychischen Störungen betroffen [25, 33], in Deutschland sind es zwischen 17 und 20 % [6, 21]. Psychische Störungen und Erkrankungen von Kindern und Jugendlichen haben in den vergangenen Jahren zugenommen [11]. Psychische Auffälligkeiten, die bereits im Kindes- und Jugendalter auftreten, belasten nicht nur die Betroffenen und ihre Familien, sondern auch das Schulsystem [21]. Bei den Kindern und Jugendlichen tritt ein Leidensdruck auf, der sich oftmals zuerst im schulischen Setting äußert [41] und von verschiedenen strukturellen Faktoren beeinflusst wird. Zu nennen sind etwa schulische Leistungserwartungen [5], immer größer werdende Klassenstrukturen [10], politische Umstrukturierungen in der Schweizer Schulentwicklung der letzten 20 Jahre [16] oder – aktuell – die Bewältigung der Coronapandemie und ihre Folgen [34, 38]. Psychische Auffälligkeiten von SchülerInnen belasten indes auch die psychische Gesundheit der Lehrpersonen [20, 21]. Als besonders belastend empfinden Lehrpersonen externalisierende Verhaltensweisen, welche sich beispielsweise in aggressivem Verhalten, Uneinsichtigkeit, Respektlosigkeit oder einer zu hohen Lautstärke äußern [26].

Schulische Präventionsprogramme im Bereich psychische Gesundheit können eine bedeutsame Symptomreduktion bei betroffenen SchülerInnen bewirken [41]. Eine frühzeitige Erkennung von Belastungen kann den Leidensdruck reduzieren [22], die Resilienz im Umgang mit Verhaltensproblemen stärken [13] und langfristigen Manifestationen psychischer Störungen entgegenwirken [2, 39]. Schulische Präventionsprogramme haben gleichzeitig auch einen positiven Effekt auf die Belastung von Lehrpersonen [41]. Während Präventionsprogramme und -maßnahmen in anderen Bereichen (beispielsweise zur Zahngesundheit) gesellschaftlich recht breit akzeptiert sind, sind Schulprogramme im Bereich der psychischen Gesundheit oftmals noch zu wenig bekannt [41] oder zu einseitig auf ein einzelnes Thema (beispielsweise Sucht) fokussiert [12].

Eine Bestandsaufnahme aus dem Jahr 2017 von Bonetti et al. [8] bezüglich Schulprogrammen in der Schweiz kommt zum Schluss, dass zwar die Mehrheit der Schweizer Volksschulen die psychische Gesundheit der Schülerschaft aktiv mit verschiedenen Programmen fördert, deren Wirksamkeit aber häufig nicht durch entsprechende Evaluationen überprüft wird. Eine Ausnahme bildet eine Zürcher Studie, die zeigen konnte, dass eine datengestützte und auf klare Ziele ausgerichtete Schulentwicklung im Rahmen von entsprechenden Schulprogrammen zu nachweisbaren Effekten geführt hat und vom Schulpersonal als entlastend wahrgenommen wurde [26]. Eine weitere Studie aus dem Raum Zürich konkludierte wiederum, dass pädagogische Fachkräfte zwar „viele verschiedene Programme und Ansätze zum Umgang mit Verhaltensauffälligkeiten und zu deren Prävention kennen und damit arbeiten“, die Situation jedoch für sie als Fachkräfte belastend bleibe [32, S. 57].

Bezogen auf den Schweizer Kontext gibt es bisher keine repräsentative Studie, die aufzeigt, wie hoch die Prävalenz an SchülerInnen mit psychischen Störungen ist, bzw. wie viele Betroffene die Lehrpersonen durchschnittlich unterrichten. Dey et al. [12] konnten für über 100 zufällig ausgewählte Berufsschulen, Fachmittelschulen und Gymnasien in der Deutschschweiz feststellen, dass die Mehrheit der befragten Lehrpersonen (> 90 %) bereits einmal eine Schülerin bzw. einen Schüler mit einer bestätigten oder vermuteten psychischen Diagnose unterrichtet haben. Dieselbe Studie wies darauf hin, dass drei Viertel der Lehrpersonen angaben, der betroffenen Schülerschaft geholfen zu haben. Bei der Art der Hilfestellung dominierte die Eigeninitiative der Lehrpersonen (beispielsweise persönliche Gespräche mit dem betroffenen Schüler, der betroffenen Schülerin gegenüber schulischen organisierten Programmen).

Der Erfolg der Umsetzung von entsprechenden Schulprogrammen ist u. a., wie Studien zeigen, maßgeblich von der psychischen Gesundheitskompetenz der Lehrpersonen abhängig [14, 27, 41, 42]. Die psychische Gesundheitskompetenz, die im Wissenschaftsdiskurs zunächst im angelsächsischen Raum als „mental health literacy“ [35] diskutiert wurde, erfasst zusätzlich zu den klassischen Dimensionen der Gesundheitskompetenz („health literacy“) – wie werden Informationen gefunden, verstanden, kritisch beurteilt und angewandt – die Erfahrungen, die Einstellungen und das Wissen in Bezug auf psychische Belastungen, Störungen und Erkrankungen. Daraus lassen sich dann wiederum Handlungsweisen eine Art Bündel an Kompetenzen im Umgang mit dem Phänomen, ableiten. Beispielsweise die Kompetenz psychische Belastungen, Störungen und Erkrankungen von SchülerInnen frühzeitig erkennen zu können, Gespräche einzuleiten oder entsprechende professionelle Fachhilfe beizuziehen. Die psychische Gesundheitskompetenz beinhaltet mit der Dimension „Informationen anwenden“ also auch die Handlung, nicht nur Wissen und Verständnis. Die „Mental-health-literacy-Bewegung“ zielte daher als wissenschaftliches Paradigma auf eine Erhöhung von Handlungskompetenzen ab [24]. Hintergrund waren eine Reihe von Untersuchungen, die gezeigt haben, dass die Erfahrungen, die Einstellungen und das Wissen über psychische Störungen von der Allgemeinbevölkerung, aber auch in spezifischen Bevölkerungsgruppen, mangelhaft war [24, 35, 40]. Dies war beispielsweise der Fall im Kontext der Erkennung von psychischen Störungen bei sich selber, aber auch bei anderen: „many members of the public cannot recognise specific disorders or different types of psychological distress“ [24, S. 396].

Bisher wurde nur wenig zu psychischer Gesundheitskompetenz von pädagogischen Lehrkräften publiziert. Almeida et al. [4] stellten in einem Sample portugiesischer Lehrpersonen eine niedrige psychische Gesundheitskompetenz fest. Ähnliches stellten Soares et al. [36] bei einer Untersuchung brasilianischer Lehrkräfte fest. Für den deutschsprachigen Raum, in welchem die Studienlage ebenfalls mager ist, zeigten Studien von Bruland et al. [9] und Hartmann et al. [19], dass pädagogische Fachkräfte oftmals überfordert sind, psychische Auffälligkeiten, Störungen oder Erkrankungen von SchüleInnen richtig einzuschätzen und im Schullalltag aufzufangen. Während die beiden deutschen Studien darauf hinweisen, dass Lehrpersonen im Umgang mit der psychischen Gesundheit von SchülerInnen unsicher sind, schätzte das Schulpersonal in der Studie von Dey et al. [12] im Schweizer Kontext die eigene psychische Gesundheitskompetenz mehrheitlich als gut oder sogar sehr gut ein, wobei es sich hierbei um eine subjektive Selbsteinschätzung der Lehrpersonen handelte.

Der aktuelle Wissenschaftsdiskurs erkennt grundsätzlich die Notwendigkeit an, die psychische Gesundheitskompetenz, und insbesondere die Einstellungen, Werte, Überzeugungen und Annahmen von Lehrpersonen in Bezug auf die psychische Gesundheit von SchülerInnen genauer zu untersuchen [17].

Mittels einer Querschnittbefragung von Lehrpersonen untersucht die vorliegende Studie als eine der ersten in der Schweiz nicht nur die Belastungssituation und Erfahrungen, sondern auch die psychische Gesundheitskompetenz von Lehrpersonen im Hinblick auf SchülerInnen mit psychischen Belastungen, Störungen und Erkrankungen:^1^

Vor diesem Hintergrund werden konkret folgende Fragestellungen fokussiert:

1. Wie hoch ist die Anzahl der von Lehrpersonen betreuten SchülerInnen mit einer psychischen Belastung, Störung oder Erkrankung (in den vergangenen 12 Monaten)?
2. Werden von Schulen und Lehrpersonen Schulprogramme zur Förderung der psychischen Gesundheit angewendet? Bestehen Unterschiede bezüglich von den Lehrpersonen selbst initiierten und auf der Basis der Schule organisierten Programmen?
3. Wie schätzen die Lehrpersonen subjektiv ihre psychische Gesundheitskompetenz ein?
4. Welches Verständnis haben Lehrpersonen über psychische Belastungen, Störungen oder Erkrankungen der Schülerschaft und inwiefern trägt dieses Verständnis (als Teil der psychischen Gesundheitskompetenz) zur Umsetzung entsprechender Programme bei?

## Methoden

Die Studie bezieht sich auf eine Querschnittbefragung von Lehrpersonen in den obligatorischen Schulstufen (Kindergarten, Primar- und Sekundarschule), die im Rahmen eines schulfinanzierten Projekts in Winterthur, einer Stadt in der deutschsprachigen Schweiz durchgeführt wurde. Bezogen auf das Schuljahr 2019/2020 wurden alle Lehrpersonen und andere im Umfeld der Schulklassen tätige pädagogische Fachkräfte (schulergänzende Betreuungsfachpersonen und Schulleitungen) zu einem Online-Survey eingeladen. Die Daten wurden anonym erhoben. Insgesamt wurden 1514 Personen für die Umfrage eingeladen. Der Feldzugang erfolgte durch die regionalen Schulbehörden. Die Datenerhebung startete im Februar 2020 und wurde Ende März 2020 aufgrund der Coronapandemie vorzeitig beendet.

Die klassischen Dimensionen der Gesundheitskompetenz wurden im Kontext der *psychischen* Gesundheitskompetenz anhand eines Instruments von Messer et al. [31] operationalisiert. Die Teilnehmenden wurden gebeten, einzuschätzen, wie einfach/schwierig es für sie ist, Informationen über psychische Belastungen, Störungen und Erkrankungen bei SchülerInnen zu finden, zu verstehen und zu beurteilen (Skala: „sehr einfach“, „ziemlich einfach“, „ziemlich schwierig“, „sehr schwierig“, „weiß nicht/keine Angabe“). Weiter wurde die Dimension der „Anwendung“ anhand verschiedener Handlungskompetenzen erfragt. Hierbei wurde ein Instrument von Ahnert et al. [1] verwendet und für den Rahmen der Studie ergänzt. Das angepasste Instrument besteht aus 17 Aussagen zu möglichen Handlungen im Umgang mit psychisch belasteten SchülerInnen (z. B.: „Ich traue mir zu, aktiv zu werden, wenn ich bei Schülerinnen und Schülern ein erhöhtes Risiko für psychische Belastung, Störung oder Erkrankung vermute“). Die Teilnehmenden konnten die 17 Aussagen jeweils auf einer 4‑Punkte-Likert-Skala beantworten („stimme überhaupt nicht zu“, „stimme eher nicht zu“, „teils/teils“, „stimme voll und ganz zu“). In der Regel entspricht der Grad der Zustimmung („stimme voll und ganz zu“) einer höheren Handlungskompetenz.

Die Lehrpersonen wurden außerdem zu Schulprogrammen im Bereich der Förderung der psychischen Gesundheit von SchülerInnen befragt. Dabei wurden Angebote und Aktivitäten auf Schulebene sowie solche, die die Lehrpersonen selbst organisiert haben, erhoben.

### Statistische Auswertung

Die Online-Umfrage wurde primär mit deskriptiven statistischen Mitteln ausgewertet. Partiell wurden Mittelwerte, Median, Standardabweichung sowie der Interquartilsabstand („interquartile range“, IQR) untersucht. Für einige Variablen wurden Häufigkeiten berechnet und bivariate Analysen angewandt, um Gruppenunterschiede (beispielsweise Primarstufe vs. Sekundarstufe) untersuchen zu können. Für eine Auswahl an Variablen wurden ebenso adjustierte Häufigkeiten berechnet, um für die unterschiedliche Verteilung bestimmter Charakteristika und deren Einfluss auf Größen wie beispielsweise die Anzahl SchülerInnen mit psychischen Problemen (letzte 12 Monate) zu korrigieren. Explorative multivariate lineare Regressionsmodelle wurden gerechnet, um für ausgewählte Variablen Zusammenhänge mit soziodemografischen und beruflichen Variablen zu untersuchen. Das Signifikanzniveau wurde bei allen Signifikanztests auf α ≤ 0,05 gesetzt. Die Auswertungen wurden mit STATA durchgeführt.

### Qualitative Auswertung

Um die psychische Gesundheitskompetenz der Lehrpersonen aus einer subjektiven Perspektive zu erfassen, wurde eine offene Frage („wie würden Sie Ihren SchülerInnen erklären, was eine psychische Störung ist“) gestellt und qualitativ ausgewertet. Die Antwortnarrationen der Befragten wurden anhand eines rekonstruierenden, inhaltsanalytischen Vorgehens [15] von zwei Forschenden aus unterschiedlichen Fachdisziplinen (Soziologie und Gesundheitsförderung) ausgewertet. Konkret wurden die Antwortnarrationen gelesen und mittels der Software MAXQDA (VERBI, Berlin, Deutschland) kodiert. Gemeinsamkeiten und v .a. Abweichungen in den Kodierungen wurden diskutiert und bei Unstimmigkeiten angepasst. Im Prozess der weiteren Datenkodierung wurden die Kodes nach den Regeln der interpretativen Sozialforschung [30] hinsichtlich Bedeutungsaspekten weiter systematisiert und so zu Hauptkodes (beispielsweise „Ursachen psychischer Erkrankungen“) und Subkodes (beispielsweise „Ursachen sind nicht-medizinisch“) verdichtet. Zur Veranschaulichung der Kodierungen wurden die direkten Zitate der Befragten im Sinn einer empirischen Verankerung beigezogen [37]. Direkte Zitate der Befragten werden in den Resultaten entsprechend kursiv und in Anführungs- und Schlusszeichen dargestellt.

## Stichprobenbeschreibung

Insgesamt haben 564 Personen die Umfrage teilweise oder komplett ausgefüllt (Rücklaufquote: 38 %). Das bereinigte Analysesample beträgt 425 Personen (Rücklaufquote: 29 %). Die Charakteristika der Stichprobe sind in Tab. 1 dargestellt. Die Zusammensetzung der teilnehmenden Lehrpersonen ist für die Lehrpersonen sämtlicher obligatorischen Schulstufen in dieser Stadt repräsentativ^2^.

**Tab. 1:** Charakteristika der Stichprobe (*N* = 425)

*Geschlecht*	*n*	*%*
Männlich	82	19,3
Weiblich	337	79,3
Möchte ich nicht sagen	6	1,4
*Alter*	*N*	*%*
30 Jahre und jünger	92	21,7
31 bis 40 Jahre	122	28,7
41 bis 50 Jahre	94	22,1
51 bis 60 Jahre	103	24,2
61 Jahre und älter	14	3,3
*Schulstufe*	*n*	*%*
Kindergarten	86	20,6
Primarschule	240	57,4
Sekundarschule	92	22,0
*Schulfunktion*	*N*	*%*
Lehrperson mit Klassenverantwortung	307	72,2
Lehrperson ohne Klassenverantwortung	73	17,2
Schulergänzende Betreuungsfachperson	35	8,2
Schulleitung mit Unterrichtsfunktion	10	2,4

Mit knapp 80 % war der überwiegende Anteil der Teilnehmenden weiblich. Die Altersstruktur war relativ gleichmäßig verteilt, wobei die größten Altersgruppen zwischen 31 und 40 Jahren (28,7 %), zwischen 41 und 50 Jahren (22,1 %) sowie zwischen 51 und 60 Jahren (24,2 %) lagen. Die Mehrheit der Teilnehmenden (57,4 %) war in Primarschulen beschäftigt, über ein Fünftel (22 %) arbeitete auf der Sekundarstufe und ein weiteres Fünftel (20,6 %) auf der Kindergartenstufe. Lehrpersonen mit Klassenverantwortung stellten mit 72,2 % die größte Schulfunktionsgruppe dar, gefolgt von Lehrpersonen ohne Klassenverantwortung (17,2 %), schulergänzenden Betreuungsfachperson (8,2 %) und Schulleitungen mit Unterrichtsfunktion (2,4 %; s. Tab. 1).

## Ergebnisse

Die meisten Teilnehmenden (94 %) gaben an, in den letzten 12 Monaten SchülerInnen mit psychischen Belastungen, Störungen und Erkrankungen betreut oder unterrichtet zu haben, durchschnittlich waren es 4,7. Ein knappes Drittel (29 %) gab 1–2 SchülerInnen an, 43 % zwischen 3 und 5, 17 % zwischen 5 und 9 und 5 % Teilnehmende gaben sogar über 10 psychisch belastete SchülerInnen an. Nur ein kleiner Anteil (6 %) betreute oder unterrichtete keine entsprechenden SchülerInnen. Die von den Teilnehmenden genannte Anzahl unterschied sich erheblich nach Schulstufe (durchschnittlich 4,7 für Kindergarten, 3,9 für Primastufen, 7,2 für Sekundarstufe) und Schulfunktion (6,4 für Lehrpersonen ohne Klassenverantwortung, 8,8 für Betreuungsfachpersonal, 12,2 für SchulleiterInnen mit Unterrichtsfunktion).

### Schulprogramme zur Förderung der psychischen Gesundheit

Auf Schulebene berichtete ein knappes Viertel (23 %) der Teilnehmenden, dass in den letzten 12 Monaten Aktivitäten und Angebote zum Thema psychische Gesundheit stattgefunden haben. Es liegen Unterschiede nach Schulstufen vor: Primarstufe: 19 %, Kindergarten: 23 %, Sekundarstufe: 33 %. Ein knappes Drittel der Teilnehmenden (31 %) gab an, im vergangenen Jahr selbst entsprechende Aktivitäten oder Angebote an ihrer Schule (beispielsweise im Klassenkontext) initiiert oder durchgeführt zu haben. Hinsichtlich der selbst initiierten Programme gab es signifikante Unterschiede nach Schulstufen (Primarstufen: 35 %, Kindergartenstufen: 23 %, Sekundarschulen: 26 %, *p* = 0,091).

Die unadjustierten Daten wiesen überdies auf einen Zusammenhang zwischen der Anzahl der belasteten SchülerInnen und den Angaben zu Schulaktivitäten hin. Teilnehmende mit mehr als 5 belasteten SchülerInnen in den vergangenen 12 Monaten berichteten häufiger (34 % gegenüber 25 % bei 3–5 SchülerInnen und 14 % bei 0–2 SchülerInnen) von entsprechend durchgeführten Programmen (auf Schulebene und selbst initiiert). Dieser Zusammenhang war sowohl für die von der Schule organisierten Programme als auch für die selbst initiierten Programme statistisch signifikant.

Die Top 3 genannten Angebote, die die Teilnehmenden auf Schulebene mit den SchülerInnen durchgeführt haben, waren Organisation von Gesundheits- oder Präventionshalbtagen (24 %), Spiel- und Sporttage (17 %) sowie Angebote im Rahmen der schulischen Sozialarbeit (18 %). Bei den selbst initiierten Angeboten waren es Achtsamkeits- und Reflexionsübungen (25 %), Gespräche mit SchülerInnen (20 %) sowie Sensibilisierung im Bereich Gefühle erkennen (8 %).

Nur knapp ein Drittel der Lehrpersonen (32 %) gab an, über genügend Tools, Lehrmittel und Angebote im Bereich der Förderung psychischer Gesundheit für die SchülerInnen zu verfügen. Auf Schulebene wünschte sich zudem knapp ein Drittel (30 %) der Teilnehmenden Weiterbildungen für sich selbst, oftmals zu spezifischen Störungsbildern (beispielsweise zu den Themen Aufmerksamkeitsdefizit-/Hyperaktivitätsstörung [ADHS] oder Trauma) sowie Unterstützung oder Empfehlungen von externen Fachpersonen aus dem medizinisch-therapeutischen Bereich. Überdies äußerten die Lehrpersonen den Wunsch, einen Leitfaden zu Thema Förderung der psychischen Gesundheit zur Hand zu haben, dem sie entnehmen können, wie sie die betroffene Schülerschaft am besten ansprechen können.

### Psychische Gesundheitskompetenz

**Abb. 1:**
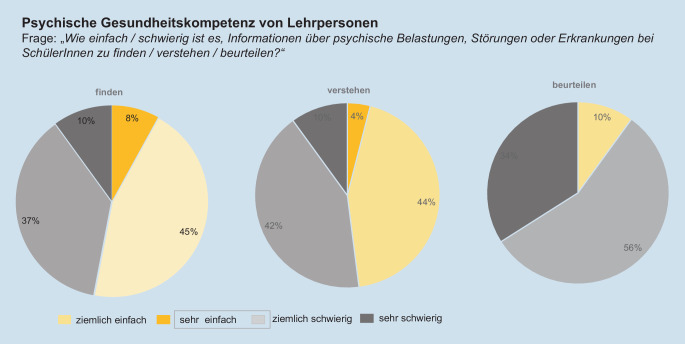
Selbsteinschätzung der Gesundheitskompetenz

Die Selbsteinschätzung der Gesundheitskompetenz der Lehrpersonen ist in Abb. 1 dargestellt. Die Mehrheit befand es zwar „ziemlich einfach“ (45 % bzw. 44 %) oder „einfach“ (8 % bzw. 4 %) Information über psychische Belastungen, Störungen und Erkrankungen *zu finden* und auch *zu verstehen*. Jedoch schätzten die Teilnehmenden es als „ziemlich schwierig“ (56 %) oder „sehr schwierig“ (34 %) ein, diese Informationen auch richtig *zu beurteilen* (z. B. im Rahmen einer konkreten Umsetzung im Klassenkontext).

**Abb. 2:**
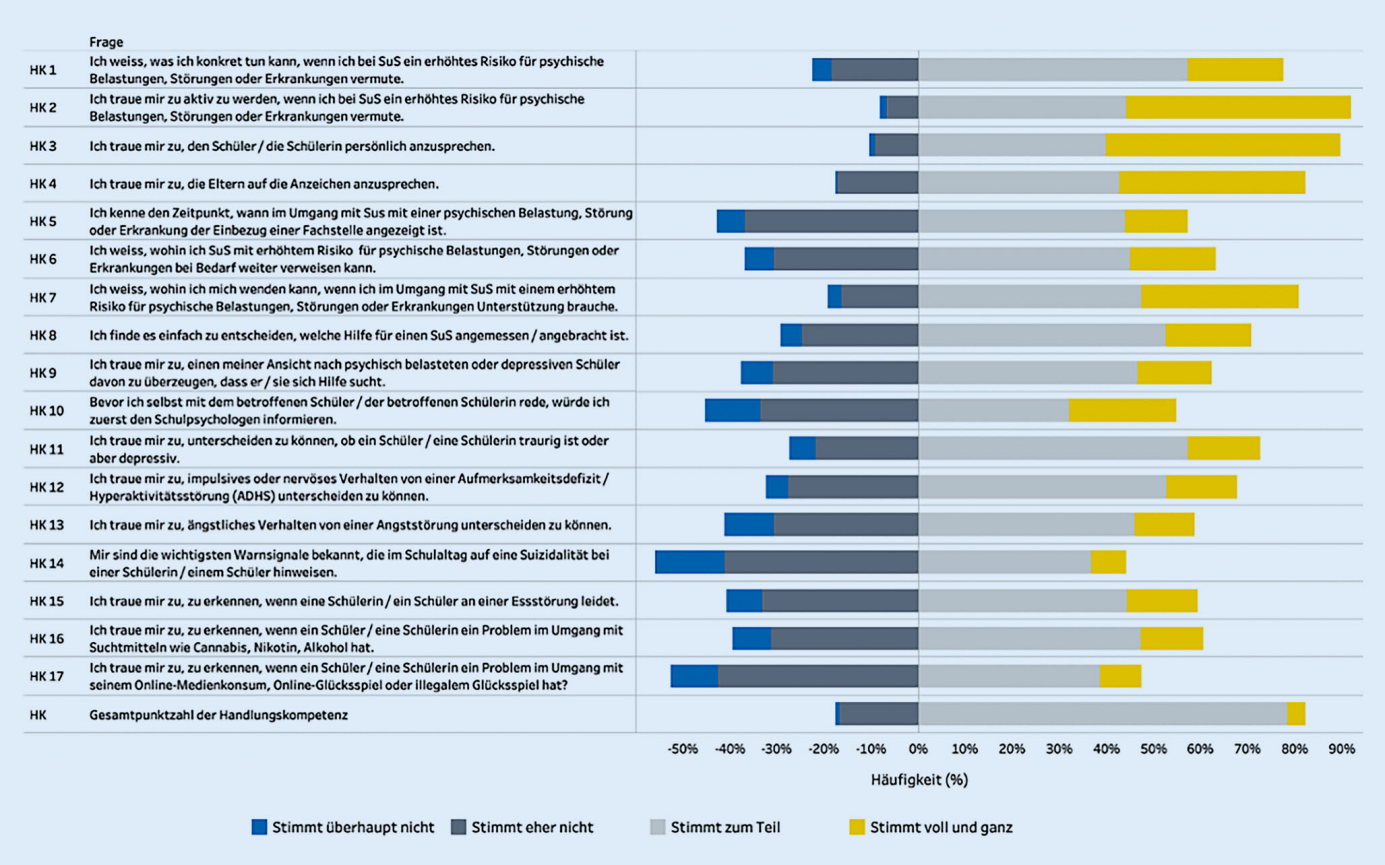
Selbsteischätzung der Handlungskompetenzen

Die Analyse der Handlungskompetenzen (Abb. 2) verdeutlichte die oben beschriebenen Tendenzen. Das betraf insbesondere die Anwendung und das Wissen der Lehrpersonen zu verschiedenen Aussagen zur Einschätzung der eigenen Handlungskompetenzen. Über alle Schulstufen hinweg wurden grundlegende, im Bereich des Schulkontexts geforderte Kompetenzen, etwa: „Ich weiß, was ich konkret tun kann, wenn ich bei Schülerinnen und Schülern ein erhöhtes Risiko für psychische Belastungen wahrnehme“ (Handlungskompetenz 1) oder „ich traue mir zu, den Schüler, die Schülerin aktiv anzusprechen“ (Handlungskompetenz 3) von den Befragten positiv eingeschätzt. Handlungskompetenzen im Zusammenhang konkreter Störungsbilder wie z. B.: „Mir sind die wichtigsten Warnsignale bekannt, die im Schullalltag auf eine Suizidalität hinweisen“ (Handlungskompetenz 14) oder „Ich traue mir zu, zu erkennen, wenn ein Schüler/eine Schülerin ein Problem im Umgang mit seinem Online-Medienkonsum, Online-Glücksspiel oder illegalem Glücksspiel hat“ (Handlungskompetenz 17) wurden als weniger machbar eingeschätzt.

### Pädagogisches Verständnis psychischer Störung

Anhand der qualitativen Analyse konnte herausdestilliert werden, dass das pädagogische Verständnis von psychischen Störungen über SchülerInnen recht divers ist. Die Lehrpersonen erwähnten die Individualität der Kinder („alle sind anders und das ist auch richtig und wichtig“), gingen aber auch auf gesellschaftliche und normative Umstände ein („gewisse Kinder handeln nicht so, wie es für uns normal oder logisch wäre“). Bei der Frage, wie sie ihren SchülerInnen erklären würden, was eine psychische Störung ist, wandten die Befragten Stilmittel, Beschreibungen und Metaphern an, die typischerweise einer pädagogischen Rhetorik und Tradition zugeordnet werden können („anhand eines Bilderbuchs oder einer Kindergeschichte“). Einige Befragte ordnen das Verhalten der betroffenen SchülerInnen entsprechend eher einem nicht-medizinischen Relevanzsystem zu, ohne dabei auf biomedizinische Ursachen einzugehen („eine Krankheit, die keine medizinische Ursache hat“). Durch diese pädagogisierende Haltung wird auffälliges Verhalten partiell relativiert („er [betroffener Schüler] denkt etwas anders als du“). Den Lehrpersonen ist es zudem wichtig, Kinder und Jugendliche mit Auffälligkeiten in der Klasse und im Unterricht zu integrieren und nicht vorschnell über sie zu urteilen („denke hier ist Inklusion schon das große Stichwort“).

Andere Befragte wiederum nahmen einen eindeutigen Bezug auf das medizinische Verständnis psychischer Störungsbilder und messen der medizinischen Diagnose eine gewisse Objektivität bei („medizinischer Krankheitsbefund wie ein Beinbruch“). Während die meisten Lehrpersonen in den Antworten dazu neigen, das psychische vom physischen Wohlbefinden zu separieren, betonen andere das „Gleichgewicht von Seele, Körper und Geist“ und dadurch auch die Notwendigkeit, medizinische Fachpersonen und -stellen in den Abklärungsprozess zu integrieren, die mehr Wissen als sie über psychische Störungen besitzen *(*„ein Problem, dass man nicht allein bewältigen kann, bei dem man Unterstützung durch eine Fachperson braucht“.).

## Diskussion

Die Mehrheit der befragten Lehrpersonen (94 %) gab an, in den letzten 12 Monaten mit psychisch belasteten SchülerInnen konfrontiert gewesen zu sein. Im Durchschnitt betreuten oder unterrichteten die Lehrkräfte knapp 5 belastete SchülerInnen.

Entsprechende Schulprogramme fanden statt, wurden aber häufiger von Lehrpersonen mittels Eigeninitiative als auf der Ebene der Schule organisiert (31 % zu 24 %). Der Großteil der Befragten hatte allerdings noch keine Erfahrungen mit entsprechenden Schulprogrammen gemacht. Dies passt zu dem Resultat, dass es den Befragten an Tools, Angeboten und Lehrmittel in diesem Bereich fehlte. Ähnlich wie in der Studie von Soares et al. [35] äußerten die Lehrkräfte den Wunsch, Weiterbildungen zu spezifischen Störungsbildern besuchen zu können. Das passt ebenfalls zum Resultat, dass es den Befragten partiell an konkretem Wissen über spezifische Handlungskompetenzen fehlte, beispielsweise im Bereich konkreter Störungsbilder, Suizidalität oder Suchtproblemen.

Die Analyse der psychischen Gesundheitskompetenz zeigt, vergleichbar mit einer anderen Studie [12], dass die Befragten eine hohe subjektive Kompetenz äußerten, Informationen zu finden und zu verstehen. An dieser Stelle ist allerdings ein „social desirability bias“ [18], eine soziale Erwünschtheit in den Antworten der Teilnehmenden auf diese Frage, nicht auszuschließen. Den Befragten unserer Studie fiel es dagegen schwerer, zu beurteilen, ob die gefunden Informationen inhaltlich korrekt sind und auf welche Weise sich diese im Schulkontext anwenden lassen. Dieser Umstand könnte dahingehend interpretiert werden, dass der Lehrplan der pädagogischen Hochschulen in gewissen Kantonen diese Kompetenzen nicht vermittelt und die Lehrpersonen in der Regel die schulnahen Dienste (beispielsweise Schulpsychologie, Schulsozialarbeit), die ressourcenabhängig die weitere Abklärung und Unterstützung für belastete SchülerInnen übernehmen, direkt involvieren. Die Lehrkräfte können zwar, wie Bonetti et al. [8] für den Deutschschweizer Kontext zeigen, oftmals auf die Unterstützung der schulinternen Dienste zählen, müssen aber selbst auch über ein notwendiges Minimum an psychischer Gesundheitskompetenz verfügen, um entsprechend an die oben erwähnten Personen zu triagieren und bei Bedarf weitere Schritte einleiten zu können. In der Umsetzung entsprechender Schulprogramme mögen ein ungenügend tiefes Verständnis [23] oder eine „Unsicherheit“ [19, S. 1173] im Umgang mit psychischen Problemen der Schülerschaft als Hindernisse gedeutet werden.

Die als mangelnd interpretierte psychische Gesundheitskompetenz im Bereich der Beurteilung könnte zudem auch auf ein genuin pädagogisches (und nicht medizinisches) Verständnis der Lehrpersonen zurückgeführt werden. Die Lehrkräfte äußerten eine Inklusionshaltung und möchten Kinder mit Auffälligkeiten inkludieren statt sie zu stigmatisieren. Auffälligkeiten wurden daher eher einem *anders-sein* zugeordnet. Die in der qualitativen Analyse beschriebene Abschwächung und Relativierung der Lehrpersonen hinsichtlich medizinsicher Ursachen mag ebenso hinderlich sein, um entsprechende Schulprogramme einzuleiten oder zu triagieren. Eine partiell ganzheitlichere Wahrnehmung psychischer Auffälligkeiten, z. B. die Berücksichtigung seelischer *und *körperlicher Zustände, wäre bedeutsam. Während eine Studie im Gegenzug zu unserer Studie ein ganzheitliches Verständnis von Lehrpersonen feststellt [36], zeigt Becker [7], dass sich pädagogische Debatten psychischer Störungen historisch tatsächlich nur teilweise an medizinischen Deutungsmuster orientieren, sich aber größtenteils eher kritisch daran abarbeiten oder die medizinische Herangehensweise zur Problematik sogar ablehnen. Ähnlich wie in der Studie von Liebsch [28] kann hier vermutet werden, dass das persönliche Verständnis und Urteil der Lehrpersonen über psychische Diagnosen konkrete und differenzierte Auswirkungen auf die Entwicklung der Schulkultur (z. B. Schulprogramme) haben. Anhand der vorliegenden qualitativen Daten kann der Zusammenhang des Verständnisses psychischer Störungen und der Implementierung entsprechender Schulprogrammen allerdings nur interpretativ hergeleitet werden. Hier bedarf es weiterer Forschungsarbeit.

Die Zunahme der Eigeninitiative der Lehrpersonen kann einerseits als Hinweis auf einen Mangel entsprechender Programme auf der Ebene der Schule gedeutet werden. Andererseits kann die Zunahme der Bedeutsamkeit einer Eigeninitiative auch im Zuge aktueller und zukünftiger gesellschaftlicher Verantwortungen betrachtet werden: Ein neuer pädagogischer Ansatz beschäftigt sich beispielswiese mit der Idee einer so genannten „futures literacy“ [3], welche nahe legt, dass Schulen die Eigeninitiative der Lehrpersonen stärken und sie ermächtigen sollen, gestärkt mit unerwarteten Veränderungen und Entwicklungen umgehen zu können.

Der Anspruch an das Wissen und die psychische Gesundheitskompetenz der Lehrpersonen nimmt zwangsläufig zu: Lehrpersonen müssen agil auf Veränderungen der Entwicklungen im Schulsystem (beispielsweise Anpassungen der Schulreformen) und gesellschaftliche Veränderungen (beispielsweise COVID-Pandemie [„coronavirus disease 2019“]) reagieren. Die partiell mangelnde psychische Gesundheitskompetenz der Lehrpersonen, insbesondere im Bereich konkreter Handlungskompetenzen, sollte auch im Sinne eines theoretischen Diskurses, wie er über Health Literacy geführt wird, keineswegs als einseitige Interaktion verstanden werden [29, 35], sondern zwingend auch strukturelle Elemente (beispielsweise die Schulentwicklungen, Schulpolitik) und gesellschaftliche Entwicklungen berücksichtigen, um die Lehrpersonen zu entlasten.

## Fazit für die Praxis

- Auf einer gesellschaftlich-politischen Ebene müssen Schulprogramme im Bereich psychische Gesundheit, ähnlich wie universelle Präventionsmaßnahmen, bekannter und zugänglicher gemacht werden.
- Auf der Ebene der Schule braucht es eine entsprechende Governance, die die Entwicklung einer Schulkultur erlaubt, welche die Bedeutung der psychischen Gesundheit im schulischen Kontext erkennt und fördert.
- Auf der Ebene der Lehrpersonen wird ein Minimum an Wissen im Bereich psychische Gesundheit benötigt (beispielsweise für die Triage). Obwohl Lehrpersonen von schulergänzenden Einrichtungen Unterstützung erhalten, sind sie oft die ersten, die mit Belastungen, Störungen und Erkrankungen von SchülerInnen direkt konfrontiert sind.
- Den Schulen wird daher empfohlen, in die Eigeninitiative der Lehrkräfte zu investieren und Weiterbildungen des Personals im Bereich psychische Gesundheit zu fördern. Die Aufgabe, das Wissen zu vermehren soll aber nicht nur bei den Lehrpersonen liegen, sondern muss auf der Ebene der Schule mit einer entsprechenden Schulkultur sowie gesamtschulpolitisch mitgetragen werden.

## Footnotes

1 In der Umfrage wurde nach psychischen Belastungen, Störungen und Erkrankungen von SchülerInnen gefragt. Ausnahme bildet die offene, qualitative Frage. Da wurde nach dem Verständnis psychischer *Störungen *gefragt.

2 Das Sample der Teilnehmenden wurde mit dem Gesamtsample des Befragungsjahrs 2019/20 der entsprechenden städtischen Schulerhebung verglichen und ist nach Alter, Geschlecht und Schulstufe repräsentativ. Für schulergänzende Betreuungsfachpersonen und weitere pädagogische Fachkräfte konnte die Repräsentativität mangels eines entsprechenden Datenvergleichs nicht überprüft werden.
